# Urotoxicity of Cyclophosphamide: A Comparison Across Neoplastic, Autoimmune, and Transplant Indications

**DOI:** 10.7759/cureus.75180

**Published:** 2024-12-05

**Authors:** Joyce H Gu, Mark Samarneh

**Affiliations:** 1 Medicine, Lake Erie College of Osteopathic Medicine, Greensburg, USA; 2 Internal Medicine/Nephrology, Riverside Health System, Yonkers, USA

**Keywords:** cyclophosphamide, disproportionality analysis, hemorrhagic cystitis, ifosfamide, urotoxicity

## Abstract

We conducted a large-scale disproportionality analysis of the urotoxicity of cyclophosphamide (CYC) and the related drug ifosfamide (IFO) using the US Food and Drug Administration Adverse Event Reporting System (FAERS) database, with data ranging from Q4 2012 to Q2 2024.

We compared the reporting odds ratio (ROR) of various urotoxicity manifestations of CYC and IFO across patient populations being treated for antineoplastic, immunosuppressive, and transplantation indications. When a wide range of urotoxicity manifestations was aggregated, we found that transplant patients had an increased relative susceptibility to CYC urotoxicity. For the notable adverse effects of chemical and viral hemorrhagic cystitis, we observed a high ROR in the overall population, but this number decreased when the population was restricted to patients treated for neoplastic or transplant indications, revealing that a significant portion of the ROR is explained by the risk associated with these indications. These conclusions did not hold among patients using the uroprotective agent MESNA, where no association with urotoxicity was found, indicating a protective effect of MESNA.

Our research reveals heterogeneous behaviors in the urotoxicity of CYC/IFO across indications and toxicity manifestations, providing healthcare professionals with valuable insights for approaching this important drug adverse event association.

## Introduction

Cyclophosphamide (CYC), a prodrug that is metabolized into cytostatic nitrogen mustard [1], is a widely used drug indicated to treat a variety of cancers and autoimmune disorders [2]. In its active form, CYC acts as a DNA alkylating agent that forms crosslinks with a cell's DNA, inhibiting DNA synthesis [1]. This cytostatic effect inhibits the proliferation of rapidly dividing cells such as cancer and immune cells, which allows its application as an antineoplastic and immunosuppressive agent. CYC is now considered a mainstay of treatment for breast cancer [3], leukemia [4], systemic lupus erythematosus (SLE) [5], vasculitis [6], and numerous other diseases. High-dose CYC has also proven useful for transplant patients as a prophylactic measure against graft-versus-host disease [7-9]. Similarly, ifosfamide (IFO), a CYC analog with a different toxicity profile, is primarily used as an antineoplastic [10].

Soon after its Food and Drug Administration (FDA) approval in 1959 [1], CYC and IFO were linked to bladder toxicity complications in both animal studies [11] and clinical studies [12], especially at higher cumulative dosages [13]. In particular, bladder toxicity manifestations including microscopic and gross hematuria [14], bladder cancer [12,15-17], acute hemorrhagic cystitis [12,16,18], bladder tamponade [18], bladder fibrosis [17,19], bladder telangiectasia [19], and increased susceptibility to bladder and urinary tract infections [20-23] have been associated with CYC and IFO. This bladder toxicity is attributed to the metabolization of CYC and IFO to the urotoxic compound acrolein [1], which incites cell injury through a variety of mechanisms including protein adduction, oxidative stress, and other effects [24]. Measures to prevent and treat such bladder and urinary tract complications include vigorous hydration and frequent voiding. The chemical agent MESNA, a compound that binds and clears acrolein [24], is effective in preventing IFO-associated urotoxicity, but clinical evidence for its use in treating CYC-associated urotoxicity remains unclear [13].

Prior studies on the urotoxicity of CYC and IFO have been largely based on individual case reports and clinical trials or retrospective analyses with limited sample sizes. To our knowledge, there has been no large-scale systematic study on the urotoxicity of CYC and IFO that encompasses multiple indications of use and manifestations of bladder toxicity, especially using data in recent years. Thus, in this work, we conducted a comprehensive study of the bladder toxicity of CYC and IFO via a disproportionality analysis of the US FDA Adverse Event Reporting System (FAERS) database. The FAERS database is used by the FDA for the post-market surveillance of drug adverse events and gathers voluntary spontaneous reports of drug adverse events. This database allows us to analyze far greater quantities of data than prior studies on the bladder toxicity of CYC/IFO and may serve as a more representative sample of the toxicity profile of CYC/IFO in real clinical practice, as opposed to controlled settings of clinical trials. In particular, by using the FAERS database, we will compare the urotoxicity characteristics of CYC/IFO across a wide variety of patient populations, such as different treatment indications, including antineoplastic, autoimmune, and transplant, as well as different urotoxicity manifestations.

## Materials and methods

Data source

We used spontaneous reports of adverse drug events provided by the FAERS database, a publicly available database published by the US FDA. Data from the fourth quarter of 2012 (2012 Q4) until the latest available data from the second quarter of 2024 (2024 Q2) are included in the study. This data is downloaded in American Standard Code for Information Interchange (ASCII) format from the FDA website and analyzed using the Python programming language.

The FAERS database is composed of various data files that capture different aspects of adverse drug event reports. We primarily used the reaction (REAC) and drug (DRUG) files, which contain information about the adverse reaction and the associated drug. These data are associated together by the primary ID (primaryid) and case ID (caseid) fields, which uniquely identify each adverse reaction report. The various brand names for CYC and IFO, such as Cytoxan, Endoxan, Ifex, and Holoxan, were identified from the database by querying the active ingredient (prod_ai) field for cyclophosphamide and ifosfamide. The resulting brand names were then used to query the drug name (drugname) field for all cases pertaining to these drugs. While there could be multiple different drug reactions associated with each case, duplicate entries were dropped by using the unique identifiers primary ID (primaryid) and case ID (caseid).

Cases about various manifestations of bladder toxicity were identified by querying the preferred term (pt) field for keywords such as “bladder,” “urinary,” “cystitis,” “haematuria,” “dysuria,” and several other relevant keywords. The preferred terms were then manually reviewed by the first author to exclude false positives from our search. Some of these false positives are relatively clear, such as “dacrocystitis” and “cholecystitis.” However, others may not be as clear and were classified at the first author’s discretion. Glucosuria, increased urine sodium, and other abnormal urinalysis results were excluded from consideration, as they are not specific to bladder toxicity. However, urinary tract infections (UTI) and findings of positive organisms in urine were included, as increased susceptibility to UTIs may occur secondary to bladder injury due to CYC and IFO [20,22]. Fields that indicate a specific cause of bladder toxicity, such as “cystitis radiation” and “BCG-related cystitis,” were also excluded.

Statistical analysis

The statistical analysis conducted in this study is based on data mining techniques for disproportionality analysis in pharmacovigilance [25]. These techniques provide quantitative measures for detecting associations between a particular drug and a particular adverse drug event. The reporting odds ratio (ROR) compares the odds of urotoxicity events among CYC/IFO adverse events and the odds of urotoxicity among all other adverse drug events.

The empirical Bayes geometric mean (EBGM) compares the percentage of urotoxicity events associated with CYC/IFO to all adverse events with this percentage, assuming that urotoxicity risk is independent of CYC/IFO use. The information component (IC2) takes the base 2 logarithm of the EBGM. The confidence interval for IC2 takes a Bayesian approach, which assumes a prior distribution for the occurrence of adverse drug events, which makes it more appropriate when the number of observations is relatively small [25]. These statistics are computed based on an incidence contingency table, as shown in Table 1.

**Table 1:** The 2 x 2 contingency table used for computation of disproportionality metrics a: The number of adverse drug event reports that report bladder toxicity associated with CYC/IFO use. b: The number of reports that report other AEs associated with CYC/IFO use. c: The number of reports that report bladder toxicity associated with other drugs. d: The number of other AEs (besides bladder toxicity) associated with other drugs (besides CYC/IFO). CYC: Cyclophosphamide; IFO: Ifosfamide; AE: Adverse event.

	AE of interest	All other AEs	Total
Drug of interest	a	b	a+b
All other drugs	c	d	c+d
Total	a+c	b+d	N = a+b+c+d

## Results

Baseline characteristics

Among the data included in the study, we identified a total of 197,185 adverse event reports associated with CYC and 20,998 adverse event reports associated with IFO. Additional demographic data, such as the distribution of the reported sex and the reported age, is presented in Table 2.

**Table 2:** Baseline characteristics of the demographic reporting adverse effects of CYC and IFO CYC: Cyclophosphamide; IFO: Ifosfamide.

Drug	Total	Sex			Age (years)				
		Female	Male	Unknown sex	<18	18-24	25-64	≥65	Unknown age
CYC	197,185	96,879	64,145	36,161	18,784	4,727	80,325	40,742	52,607
IFO	20,998	6,650	9,624	4,724	4,292	1,313	6,882	1,897	6,614

Among the bladder toxicity reports that we identify, the 10 adverse effects with highest incidence were urinary tract infection (n = 1,728), hemorrhagic cystitis (n = 1,422), hematuria (n = 786), cystitis (n = 496), urinary retention (n = 342), dysuria (n = 310), viral hemorrhagic cystitis (n = 293), urinary incontinence (n = 228), *Escherichia *urinary tract infection (n = 211), and pollakiuria (frequent daytime urination) (n = 182). It is important to note that some of the preferred terms reported by the FAERS database may be overlapping or redundant (e.g., urinary tract infection and *Escherichia *urinary tract infection), which may make the use of the preferred terms themselves a suboptimal grouping of the adverse event reports. We later rectified this problem with a more refined analysis.

Aggregated disproportionality analysis

We first conducted a disproportionality analysis that aggregated all patients and all bladder toxicity manifestations together. Among our dataset, we identified 6,276 reports of urotoxicity adverse events associated with CYC and 530 reports of urotoxicity adverse events associated with IFO. We also conducted a disproportionality analysis for the 10 adverse events with the highest incidence when combined across CYC and IFO. The full statistics are available in Table 3.

**Table 3:** Disproportionality analysis of the bladder toxicity of cyclophosphamide and ifosfamide a: The number of adverse drug event reports that report bladder toxicity associated with CYC/IFO use. b: The number of reports that report other AEs associated with CYC/IFO use. c: The number of reports that report bladder toxicity associated with other drugs. d: The number of other AEs (besides bladder toxicity) associated with other drugs (besides CYC/IFO). ROR: Reporting odds ratio; IC2: Information component; CI: Confidence interval; CYC: Cyclophosphamide; IFO: Ifosfamide.

Drug	Adverse event	a	b	c	d	ROR [95% CI]	IC2 [95% CI]
CYC	Bladder toxicity	6,276	182,640	453,754	16,640,063	1.26 [1.23, 1.29]	0.32 [0.28, 0.36]
IFO	Bladder toxicity	530	20,307	459,500	16,802,396	0.95 [0.88, 1.04]	-0.07 [-0.19, 0.06]
CYC	Cystitis	447	188,469	32,289	17,061,528	1.25 [1.14, 1.38]	0.32 [0.18, 0.46]
	Hemorrhagic cystitis	1,286	187,630	1,972	17,091,845	59.40 [55.37, 63.74]	5.14 [5.04, 5.23]
	Dysuria	289	188,627	31,688	17,062,129	0.82 [0.73, 0.93]	-0.27 [-0.44, -0.10]
	Escherichia UTI	189	188,727	3,721	17,090,096	4.60 [3.97, 5.32]	2.12 [1.90, 2.33]
	Hematuria	697	188,219	29,643	17,064,174	2.13 [1.98, 2.30]	1.07 [0.96, 1.18]
	Pollakiuria	171	188,745	36,262	17,057,555	0.43 [0.37, 0.50]	-1.21 [-1.44, -0.99]
	Urinary incontinence	189	188,727	24,891	17,068,926	0.69 [0.60, 0.79]	-0.53 [-0.74, -0.32]
	Urinary retention	286	188,630	27,155	17,066,662	0.95 [0.85, 1.07]	-0.07 [-0.24, 0.10]
	UTI	1,637	187,279	162,525	16,931,292	0.91 [0.87, 0.96]	-0.13 [-0.20, -0.06]
	Viral hemorrhagic cystitis	268	188,648	319	17,093,498	76.12 [64.71, 89.55]	5.18 [4.97, 5.39]
IFO	Cystitis	21	20,816	32,715	17,229,181	0.53 [0.35, 0.82]	-0.88 [-1.50, -0.26]
	Hemorrhagic cystitis	129	20,708	3,129	17,258,767	34.36 [28.80, 41.00]	4.72 [4.46, 4.98]
	Dysuria	18	20,819	31,959	17,229,937	0.47 [0.29, 0.74]	-1.06 [-1.72, -0.40]
	Escherichia UTI	21	20,816	3,889	17,258,007	4.48 [2.91, 6.88]	1.94 [1.33, 2.56]
	Hematuria	77	20,760	30,263	17,231,633	2.11 [1.69, 2.64]	1.05 [0.73, 1.38]
	Pollakiuria	9	20,828	36,424	17,225,472	0.20 [0.11, 0.39]	-2.17 [-3.08, -1.25]
	Urinary incontinence	36	20,801	25,044	17,236,852	1.19 [0.86, 1.65]	0.24 [-0.23, 0.72]
	Urinary retention	46	20,791	27,395	17,234,501	1.39 [1.04, 1.86]	0.46 [0.04, 0.89]
	UTI	74	20,763	164,088	17,097,808	0.37 [0.30, 0.47]	-1.41 [-1.74, -1.07]
	Viral hemorrhagic cystitis	12	20,825	575	17,261,321	17.30 [9.76, 30.64]	2.93 [2.12, 3.74]

When all the bladder manifestations are aggregated together, only a mild association was observed between CYC and bladder toxicity (ROR = 1.26, 95% CI: 1.23, 1.29), and no association was observed between IFO and bladder toxicity (ROR = 0.95, 95% CI: 0.88, 1.04). When examining the breakdown for the top 10 adverse effects by incidence, the increased association generally comes from the more severe manifestations of bladder toxicity such as chemical hemorrhagic cystitis and viral hemorrhagic cystitis, while less severe manifestations such as dysuria, pollakiuria, and urinary retention do not exhibit an increased association. In particular, the ROR for hemorrhagic cystitis is ROR = 59.40 (95% CI: 55.37, 63.74) for CYC and ROR = 34.36 (95% CI: 28.80, 41.00), and the ROR for viral hemorrhagic cystitis is ROR = 76.12 (95% CI: 64.71, 89.55) for CYC and ROR = 17.30 (95% CI: 9.76, 30.64) for IFO. Thus, one conclusion to be drawn is that in the overall population, CYC and IFO increase the risk of severe bladder toxicity events such as hemorrhagic cystitis, while the risk of bladder toxicity, in general, is substantially less prominent.

While the aggregated analysis provides some initial insights, there are additional factors that must be considered when interpreting these results. For instance, as described previously, the preferred terms used to report these adverse effects are often redundant; for example, *Escherichia *UTI is treated as a different adverse effect from UTI according to the classification in the FAERS database, which is not a meaningful separation in this context and perhaps should be treated together. Another issue is that in some cases, the underlying indication for CYC or IFO therapy itself may be implicated as a risk factor for bladder toxicity. For example, viral hemorrhagic cystitis has been increasingly recognized as a complication of transplants, both in solid organ transplantation and stem cell transplantation [23,26-28]. Therefore, it is important to distinguish between the bladder toxicity risk associated with transplantation and the bladder toxicity risk associated with cyclophosphamide used to treat the transplant. Finally, a third factor that may greatly influence the bladder toxicity risk of CYC/IFO is whether the patient has been given concurrent MESNA therapy, due to the uroprotective effects of MESNA [24]. These possible confounding factors necessitate a stratified analysis that controls these differences to capture the idiosyncratic bladder toxicity risks associated with CYC/IFO itself.

Stratified analysis by indication

Our first stratified analysis considers a categorization of the indication of CYC and IFO usage. The indications for CYC can be largely classified as either a neoplastic disorder, an autoimmune disorder, or transplant complication treatment and prophylaxis. We first classified 97.2% of the adverse drug event cases pertaining to CYC or IFO as either one of these cases or explicitly excluded. Some indications in the category of neoplastic disorders include breast cancer and Hodgkin's disease; some indications in the category of autoimmune disorders include systemic lupus erythematosus and Wegener's granulomatosis; some indications in the category of transplant include allogenic bone marrow transplantation therapy and graft versus disease; and some indications that were explicitly excluded include unknown indication, accidental exposure, and non-specific classifications, which could encompass a variety of indications such as immunosuppression and prophylaxis. The most common indication for CYC according to our classification was neoplastic disorders with 3,963 reports associated with these disorders, followed by transplant therapy (n = 1,528) and autoimmune disorders (n = 696). For IFO, the vast majority of adverse events were reported for neoplastic treatments (n = 472), with only a handful of reports associated with autoimmune disorder treatment (n = 11) and transplant therapy (n = 40). The full set of results are available in Table 4.

**Table 4:** Disproportionality analysis of the bladder toxicity of cyclophosphamide and ifosfamide, stratified by indication of use. Results are omitted when there are zero reports of adverse events associated with the drug. a: The number of adverse drug event reports that report bladder toxicity associated with CYC/IFO use. b: The number of reports that report other AEs associated with CYC/IFO use. c: The number of reports that report bladder toxicity associated with other drugs. d: The number of other AEs (besides bladder toxicity) associated with other drugs (besides CYC/IFO). ROR: Reporting Odds ratio; IC2: Information component; CI: Confidence interval; CYC: Cyclophosphamide; IFO: Ifosfamide.

Drug	Indication	a	b	c	d	ROR [95% CI]	IC2 [95% CI]
Bladder toxicity							
CYC	Antineoplastic	3,963	144,283	59,393	2,047,293	0.95 [0.92, 0.98]	-0.07 [-0.12, -0.02]
	Autoimmune	696	13,216	79,202	1,618,419	1.08 [1.00, 1.16]	0.10 [-0.01, 0.21]
	Transplant	1,528	15,468	5,006	97,714	1.93 [1.82, 2.05]	0.72 [0.64, 0.80]
IFO	Antineoplastic	472	18,279	62,884	2,173,297	0.89 [0.81, 0.98]	-0.16 [-0.29, -0.02]
	Autoimmune	11	173	79,887	1,631,462	1.30 [0.71, 2.39]	0.32 [-0.54, 1.18]
	Transplant	40	789	6,494	112,393	0.88 [0.64, 1.21]	-0.18 [-0.64, 0.29]
Hemorrhagic cystitis							
CYC	Antineoplastic	537	147,709	584	2,106,102	13.11 [11.66, 14.74]	2.85 [2.70, 3.00]
	Autoimmune	63	13,849	139	1,697,482	55.55 [41.23, 74.85]	4.59 [4.18, 5.01]
	Transplant	825	16,171	482	102,238	10.82 [9.66, 12.12]	2.15 [2.02, 2.27]
IFO	Antineoplastic	124	18,627	997	2,235,184	14.92 [12.38, 18.00]	3.60 [3.32, 3.87]
	Transplant	9	820	1,298	117,589	0.99 [0.51, 1.92]	-0.01 [-0.93, 0.91]
Viral hemorrhagic cystitis							
CYC	Antineoplastic	136	148,110	59	2,106,627	32.79 [24.15, 44.50]	3.30 [2.98, 3.62]
	Autoimmune	17	13,895	16	1,697,605	129.81 [65.57, 256.97]	3.82 [2.98, 4.66]
	Transplant	185	16,811	238	102,482	4.74 [3.91, 5.75]	1.60 [1.35, 1.86]
IFO	Antineoplastic	11	18,740	184	2,235,997	7.13 [3.88, 13.11]	2.19 [1.33, 3.05]
	Transplant	10	819	413	118,474	3.50 [1.86, 6.58]	1.48 [0.59, 2.37]

The analysis reveals that transplant patients being treated with CYC demonstrate a significant elevation in bladder toxicity over the risk in the overall population, with ROR = 1.93 (95% CI: 1.82, 2.05). Thus, our results indicate that the population of transplant patients may be more susceptible to CYC-induced bladder toxicity.

On the other hand, for patients using CYC to treat neoplastic disorders and autoimmune disorders, there was no significant association with bladder toxicity risk, with ROR = 0.95 (95% CI: 0.92, 0.98) and ROR = 1.08 (95% CI: 1.00, 1.16), respectively. Similarly, patients using IFO do not exhibit a significant association with bladder toxicity risk across the three categories of indication, with ROR = 0.89 (95% CI: 0.81, 0.98) for neoplastic disorders, ROR = 1.30 (95% CI: 0.71, 2.39) for autoimmune disorders, and ROR = 0.88 (95% CI: 0.64, 1.21) for transplant complication prophylaxis.

We also examined the effect of stratification on the prominent adverse effects of hemorrhagic cystitis and viral hemorrhagic cystitis in particular. For hemorrhagic cystitis seen with CYC use, we observed that under autoimmune indications, the ROR of hemorrhagic cystitis did not differ significantly from the overall population (ROR = 55.55, 95% CI: 41.23, 74.85), indicating stability of our conclusions. On the other hand, under neoplastic and transplant indications, the ROR is still significant but substantially less so, with ROR = 13.11 (95% CI: 11.66, 14.74) and ROR = 10.82 (95% CI: 9.66, 12.12), respectively. Thus, the analysis reveals that the high risk of hemorrhagic cystitis is partially explained by a high risk of this adverse effect in the population of patients being treated for neoplasms and transplantations. However, CYC is still an independent risk factor for hemorrhagic cystitis even in this population, with roughly a 10-fold increase in the odds among patients being treated for neoplasms and transplants. For IFO, we again noted that the treatment indication partially explained the high odds ratio. IFO has a ROR of 14.92 (95% CI: 12.38, 18.00) among patients with neoplastic indications, while there was no association among transplant patients (ROR = 0.99, 95% CI: 0.51, 1.92), and no reports on hemorrhagic cystitis were seen with IFO treatment for autoimmune indications.

Similar effects are observed with viral hemorrhagic cystitis, where for CYC, the ROR for viral hemorrhagic cystitis is significant yet lower among transplant patients (ROR = 4.74, 95% CI: 3.91, 5.75), indicating that a substantial part of viral hemorrhagic cystitis risk is explained by transplantation itself. On the other hand, among neoplastic and autoimmune indications, the ROR remains high with ROR = 32.79 (95% CI: 24.15, 44.50) and ROR = 129.81 (95% CI: 65.57, 256.97), respectively. IFO carries a significant yet decreased ROR among neoplastic (ROR = 713, 95% CI: 3.88, 13.11) and transplant (ROR = 3.50, 95% CI: 1.86, 6.58) indications, and there are no reports of viral hemorrhagic cystitis seen with IFO treatment for autoimmune indications.

Stratified analysis by bladder toxicity manifestation

Next, we conducted a disproportionality analysis that categorized bladder toxicity manifestations into categories. We aimed to control the differences in these manifestations of bladder toxicity without getting distracted by minor differences in the adverse event reporting (e.g., *Escherichia *UTI versus UTI) and without excluding rare adverse events such as bladder cancer. We considered four categories: (1) one encompassing bladder cancers and neoplasms, (2) the second one encompassing bladder hemorrhage including hematuria and hemorrhagic cystitis, (3) the third encompassing bladder and UTI, and (4) the last one encompassing other miscellaneous events such as oliguria, dysuria, pyuria, and noninfective cystitis. The full set of results is presented in Table 5.

**Table 5:** Disproportionality analysis of the bladder toxicity of cyclophosphamide and ifosfamide, stratified by bladder toxicity manifestation and indication of use. Results are omitted when there zero reports of adverse events associated with the drug. a: The number of adverse drug event reports that report bladder toxicity associated with CYC/IFO use. b: The number of reports that report other AEs associated with CYC/IFO use. c: The number of reports that report bladder toxicity associated with other drugs. d: The number of other AEs (besides bladder toxicity) associated with other drugs (besides CYC/IFO). ROR: Reporting Odds ratio; IC2: Information component; CI: Confidence interval; CYC: Cyclophosphamide; IFO: Ifosfamide.

Drug	Indication	a	b	c	d	ROR [95% CI]	IC2 [95% CI]
Bladder cancer							
CYC	Antineoplastic	126	148,120	2,084	2,104,602	0.86 [0.72, 1.03]	-0.20 [-0.47, 0.06]
	Autoimmune	34	13,878	1,824	1,695,797	2.28 [1.62, 3.20]	1.12 [0.63, 1.61]
	Transplant	20	16,976	102	102,618	1.19 [0.73, 1.91]	0.19 [-0.50, 0.87]
IFO	Antineoplastic	3	18,748	2,207	2,233,974	0.16 [0.05, 0.50]	-2.28 [-3.72, -0.83]
Bladder hemorrhage							
CYC	Antineoplastic	954	147,292	9,690	2,096,996	1.40 [1.31, 1.50]	0.45 [0.35, 0.54]
	Autoimmune	258	13,654	5,388	1,692,233	5.93 [5.23, 6.73]	2.47 [2.28, 2.65]
	Transplant	945	16,051	1,112	101,608	5.38 [4.92, 5.88]	1.69 [1.58, 1.80]
IFO	Antineoplastic	189	18,562	10,455	2,225,726	2.17 [1.88, 2.50]	1.09 [0.87, 1.30]
	Transplant	19	810	2,038	116,849	1.34 [0.85, 2.12]	0.39 [-0.27, 1.05]
Bladder infection							
CYC	Antineoplastic	1,925	146,321	27,140	2,079,546	1.01 [0.96, 1.06]	0.01 [-0.06, 0.08]
	Autoimmune	326	13,586	46,884	1,650,737	0.84 [0.76, 0.94]	-0.23 [-0.40, -0.07]
	Transplant	520	16,476	2,953	99,767	1.07 [0.97, 1.17]	0.08 [-0.06, 0.21]
IFO	Antineoplastic	127	18,624	28,938	2,207,243	0.52 [0.44, 0.62]	-0.92 [-1.18, -0.67]
	Autoimmune	11	173	47,199	1,664,150	2.24 [1.22, 4.12]	0.98 [0.12, 1.84]
	Transplant	20	809	3,453	115,434	0.83 [0.53, 1.29]	-0.26 [-0.90, 0.38]
Other							
CYC	Antineoplastic	1,400	146,846	26,608	2,080,078	0.75 [0.71, 0.79]	-0.40 [-0.47, -0.32]
	Autoimmune	181	13,731	35,160	1,662,461	0.62 [0.54, 0.72]	-0.66 [-0.88, -0.45]
	Transplant	203	16,793	1,509	101,211	0.81 [0.70, 0.94]	-0.26 [-0.47, -0.05]
IFO	Antineoplastic	191	18,560	27,817	2,208,364	0.82 [0.71, 0.94]	-0.28 [-0.49, -0.07]
	Transplant	14	815	1,698	117,189	1.19 [0.70, 2.02]	0.22 [-0.53, 0.98]

In the category of bladder cancers, patients treated for autoimmune disorders with CYC showed an increased association (ROR = 2.28, 95% CI: 1.62, 3.20). In contrast, patients treated for neoplastic disorders (ROR = 0.86, 95% CI: 0.72, 1.03) and transplants (ROR = 1.19, 95% CI: 0.73, 1.91) do not exhibit an association. Thus, the results indicate that the risk of developing bladder cancer is most prominent when the patient is treated for an autoimmune disorder, whereas the bladder cancer risk is more appropriately attributed to the underlying treatment indication among patients treated for neoplasms or transplants. For IFO, bladder cancer is rare, which has been noted in prior literature [17].

In the category of bladder hemorrhage, which includes both severe forms such as hemorrhagic cystitis and precursor signs such as microscopic and gross hematuria, the risk associated with CYC use is still significantly elevated in the population of autoimmune (ROR = 5.93, 95% CI: 5.23, 6.73) and transplant (ROR = 5.38, 95% CI: 4.92, 5.88) indications, while this risk diminishes for neoplastic indications (ROR = 1.40, 95% CI [1.31, 1.50]). IFO exhibits a statistically similar ROR for neoplastic indications (ROR = 2.17, 95% CI: 1.88, 2.50) and has no significant association with transplant indications (ROR = 1.34, 95% CI: 0.85, 2.12).

Our stratified analysis suggests that the adverse event associations for bladder infection and other bladder toxicity manifestations are largely unremarkable.

Finally, we investigate the effects of stratifying the analysis by using MESNA, a uroprotective agent frequently prescribed along with CYC/IFO. We present results only for the adverse events identified as prominent in the prior sections. The complete results are presented in Table 6.

**Table 6:** Disproportionality analysis of the bladder toxicity of cyclophosphamide and ifosfamide, stratified by bladder toxicity manifestation and MESNA use. Results are omitted when there are zero reports of adverse events associated with the drug. a: The number of adverse drug event reports that report bladder toxicity associated with CYC/IFO use. b: The number of reports that report other AEs associated with CYC/IFO use. c: The number of reports that report bladder toxicity associated with other drugs. d: The number of other AEs (besides bladder toxicity) associated with other drugs (besides CYC/IFO). ROR: Reporting Odds ratio; IC2: Information component; CI: Confidence interval; CYC: Cyclophosphamide; IFO: Ifosfamide.

Drug	MESNA	Indication	a	b	c	d	ROR [95% CI]	IC2 [95% CI]
Bladder cancer								
CYC	No	Antineoplastic	126	146,730	2,084	2,103,850	0.87 [0.72, 1.04]	-0.19 [-0.46, 0.07]
		Autoimmune	34	13,793	1,824	1,695,790	2.29 [1.63, 3.22]	1.13 [0.63, 1.62]
		Transplant	20	16,697	102	102,602	1.20 [0.75, 1.95]	0.20 [-0.48, 0.89]
IFO	No	Antineoplastic	3	17,690	2,207	2,232,890	0.17 [0.06, 0.53]	-2.20 [-3.64, -0.75]
Bladder hemorrhage								
CYC	No	Antineoplastic	932	145,924	9,673	2,096,261	1.38 [1.29, 1.48]	0.43 [0.33, 0.53]
		Autoimmune	253	13,574	5,388	1,692,226	5.85 [5.15, 6.65]	2.45 [2.26, 2.63]
		Transplant	918	15,799	1,111	101,593	5.31 [4.86, 5.81]	1.69 [1.57, 1.80]
	Yes	Antineoplastic	22	1,368	17	735	0.70 [0.37, 1.32]	-0.23 [-0.98, 0.52]
		Autoimmune	5	80	0	7	--	--
		Transplant	27	252	1	15	1.61 [0.20, 12.65]	-0.01 [-0.74, 0.72]
IFO	No	Antineoplastic	174	17,519	10,431	2,224,666	2.12 [1.82, 2.46]	1.05 [0.83, 1.27]
		Transplant	19	792	2,010	116,600	1.39 [0.88, 2.20]	0.43 [-0.22, 1.09]
	Yes	Antineoplastic	15	1,043	24	1,060	0.64 [0.33, 1.22]	-0.37 [-1.23, 0.48]
Hemorrhagic cystitis								
CYC	No	Antineoplastic	528	146,328	571	2,105,363	13.30 [11.82, 14.98]	2.86 [2.71, 3.02]
		Autoimmune	60	13,767	139	1,697,475	53.22 [39.30, 72.08]	4.54 [4.12, 4.97]
		Transplant	809	15,908	481	102,223	10.81 [9.64, 12.11]	2.16 [2.03, 2.29]
	Yes	Antineoplastic	9	1,381	13	739	0.37 [0.16, 0.87]	-0.67 [-1.76, 0.42]
		Autoimmune	3	82	0	7	--	--
		Transplant	16	263	1	15	0.91 [0.11, 7.35]	-0.07 [-1.02, 0.88]
IFO	No	Antineoplastic	112	17,581	987	2,234,110	14.42 [11.85, 17.54]	3.55 [3.27, 3.84]
		Transplant	9	802	1,281	117,329	1.03 [0.53, 1.99]	0.03 [-0.89, 0.95]
	Yes	Antineoplastic	12	1,046	10	1,074	1.23 [0.53, 2.86]	0.07 [-0.93, 1.07]
Viral hemorrhagic cystitis								
CYC	No	Antineoplastic	136	146,720	59	2,105,875	33.08 [24.37, 44.91]	3.31 [2.99, 3.64]
		Autoimmune	17	13,810	16	1,697,598	130.61 [65.98, 258.55]	3.82 [2.98, 4.66]
		Transplant	185	16,532	238	102,466	4.82 [3.97, 5.84]	1.62 [1.37, 1.88]
IFO	No	Antineoplastic	11	17,682	184	2,234,913	7.56 [4.11, 13.89]	2.24 [1.38, 3.10]
		Transplant	10	801	413	118,197	3.57 [1.90, 6.72]	1.50 [0.62, 2.39]

Our analysis reveals that MESNA indeed has a significant effect on the urotoxicity. First, we noted that no cases of bladder cancer were reported as an adverse effect of CYC or IFO when MESNA is used concomitantly. However, this may partially be explained by a small number of patients who use MESNA: 2,142 patients using MESNA being treated for neoplasms, 92 patients using MESNA being treated for autoimmune disorders, and 295 patients using MESNA being treated for transplants. For bladder hemorrhage, there are a few reports of this adverse event even with MESNA use, but the ROR remains significantly lower in the population of patients using MESNA compared to those not using MESNA. Similar conclusions can be drawn for bladder toxicity adverse events, such as hemorrhagic cystitis and viral hemorrhagic cystitis. Among patients who do not use MESNA, the ROR results are consistent with those obtained without stratification by MESNA use. However, in patients using MESNA, there is no association with bladder toxicity.

## Discussion

In this study, we conducted a disproportionality analysis of the bladder toxicity of CYC and its analog IFO using data provided by the FAERS database. Various forms of bladder toxicity including bladder cancer [12,14-15,17], hemorrhagic cystitis [12-13,18], bladder infection [20,22-23], and other manifestations [19] have long affected patients using CYC and IFO; therefore, it is critical to investigate and understand these effects, especially in patients who are at greater risk.

We first conducted an aggregated disproportionality analysis of the entire population, which showed that CYC had an aggregate ROR of 1.26 (95% CI: 1.23, 1.29) for bladder toxicity, and IFO had an aggregate ROR of 0.95 (95% CI: 0.88, 1.04) for bladder toxicity (Table 3). Among the top 10 adverse events with the highest incidence, hemorrhagic cystitis and viral hemorrhagic cystitis have a markedly increased ROR (Table 3). Stratification of this analysis by the indication of use (antineoplastic, autoimmune, or transplantation) for CYC/IFO reveals that the ROR for hemorrhagic cystitis and viral hemorrhagic cystitis remains elevated, although to a lesser degree at times (Table 4), which indicates that the high ROR can be partially explained by the underlying indication for use. However, the ROR for bladder toxicity overall is increased in transplant patients, which may be attributed to the use of higher dosages for these treatments (Table 4). Thus, clinicians should consider transplant patients to be a higher-risk population for developing urotoxicity symptoms and evaluate them for bladder injury via cystoscopy more frequently. When the adverse events are grouped into categories of bladder toxicity manifestations of bladder cancer, bladder hemorrhage, bladder infection, and other manifestations, bladder cancer and bladder hemorrhage exhibit statistically significant increases in ROR, which also vary when stratified for the underlying indication for CYC/IFO use (Table 5). Finally, when stratified by concomitant MESNA use, the analysis reveals a protective effect of MESNA: The increased ROR is found only in patients who do not use MESNA and not in patients who do (Table 6). Thus, our findings support the usage of MESNA in patients who are at a higher risk of bladder injury from the use of CYC/IFO.

One novel aspect of this study is that the use of the FAERS database provides a large sample size to compare the bladder toxicity risks across different indications for treatment. This allows for the distinction between the bladder toxicity risk associated with a given underlying condition, such as neoplasms or transplantation, and the bladder toxicity risk associated with the pharmacotherapy for this condition. For example, transplantation itself is known to pose an increased risk of viral hemorrhagic cystitis due to adenovirus and BK virus [23,26-28]. Therefore, an increased ROR for viral hemorrhagic cystitis associated with CYC may be due to its association with transplantation treatment. Our results indicate that this is partially the case, but even among transplant patients, CYC still has a significantly elevated ROR for viral hemorrhagic cystitis (Table 4). This may be explained by the fact that, when compared to other transplant pharmacotherapies, CYC has multiple potential mechanisms for inciting viral hemorrhagic cystitis, including the immunosuppressive effect of CYC, which increases susceptibility to infections, as well as the direct bladder injury due to acrolein. We conducted similar analyses for overall bladder toxicity and hemorrhagic cystitis (Table 4), and other categories of bladder toxicity manifestations (Table 5).

When comparing the overall risk of bladder toxicity across different indications, transplant patients exhibit a higher risk, with an ROR of 1.93 (95% CI: 1.82, 2.05) compared to 1.26 (95% CI: 1.23, 1.29) overall (Table 4). This may reflect the increased susceptibility of these patients to CYC-induced bladder toxicity.

Surprisingly, few prior studies have demonstrated the uroprotective effect of MESNA for preventing hemorrhagic cystitis in clinical settings. The only relevant study identified is by Yilmaz et al. [13], which reported that MESNA did not provide a protective effect in a study of 1,018 patients treated with CYC for rheumatic disease [13,29]. However, the present study, involving a much larger population, demonstrates a significant protective effect of MESNA against hemorrhagic cystitis (Table 6).

Although the FAERS database enables an analysis of a very large sample of patients, it is also associated with several disadvantages that pose limitations for this study. It is generally accepted that findings from investigations of the FAERS database should be considered as "flags," which should be confirmed by other study designs that will ensure generalizability to clinical settings [25]. First, the FAERS database contains only reports that are positive for adverse effects, which inherently selects for a population that may be significantly different from the general population (e.g., overall higher sensitivity to drug reactions and higher likelihood of having other risk factors). Thus, it would be of interest to verify the findings of the present study in a sample that does not have such a selection bias, such as in the setting of clinical trials. Such clinical studies would also be able to investigate longer-term characteristics of these adverse events with more detail on the progression and persistence of these effects. The FAERS database is also limited to adverse event reports that are reported spontaneously, which introduces another layer of possible bias, which would also be rectified by repeating this study in a more controlled setting. Some factors that may influence the spontaneous reporting of these events may include treatment indications, patient and/or physician awareness of adverse event reporting systems, or socioeconomic factors. Our analysis may also benefit from further stratification, such as investigating the difference between solid organ and hematopoietic cell transplants. Other factors to be considered additionally include dosages and the severity of the adverse events. These areas warrant further investigation in future studies.

## Conclusions

In this study, we analyzed the urotoxicity of CYC and IFO using a disproportionality analysis approach and specifically compared the urotoxicity of these drugs across patients treated for different categories of indications. Our research reveals heterogeneous behaviors in the urotoxicity of CYC/IFO across various indications and toxicity manifestations, providing healthcare professionals valuable insights for approaching this important drug adverse event association.
